# A Small-Volume Apparatus for the Measurement of Phase Equilibria

**DOI:** 10.6028/jres.109.039

**Published:** 2004-12-01

**Authors:** Stephanie L. Outcalt, Byung-Chul Lee

**Affiliations:** National Institute of Standards and Technology, Boulder, CO 80305-3328

**Keywords:** butane, phase equilibria, propane, propane + butane, vapor pressure

## Abstract

An apparatus has been designed and constructed for the measurement of vapor-liquid equilibrium properties. The main components of the apparatus consist of an equilibrium cell and a vapor circulation pump. The cell and all of the system valves are housed inside a temperature controlled, insulated aluminum block. The temperature range of the apparatus is 260 K to 380 K to pressures of 6 MPa. The uncertainty of the temperature measurement is 0.03 K, and the uncertainty in the pressure measurement is 9.8 × 10^−4^ MPa. An automated data acquisition system is used to measure temperature and pressure at equilibrium. The apparatus has been performance tested by measuring the vapor pressures of propane, butane, and a standard mixture of propane + butane.

## 1. Introduction

Phase equilibria data are some of the cornerstones necessary to develop equations of state. A main focus of our research group is to measure thermophysical properties of fluids in order to facilitate the development of such equations. A prime design objective of the present apparatus is the small volume of the equilibrium system. The small volume of this apparatus makes it attractive for measurements on hazardous materials where safety is of particular concern, or when it is difficult or expensive to acquire large amounts of a particular fluid. The second major design decision was to eliminate the sampling and composition analysis that complicate many vapor-liquid equilibrium measurements. This requires the preparation of standard mixtures of known composition, but reduces the major uncertainty of composition and permits a simpler, more automated design.

The main components of the apparatus are the equilibrium cell and a vapor circulation pump. An automated data acquisition system is used to collect equilibrium measurements of temperature and pressure. Systems with multiple components can be studied by preparing a gas phase standard mixture consisting of the components of interest, and then condensing it into the system leaving only a very small vapor space. In this manner, vapor pressures of a fixed composition liquid are measured.

## 2. Experimental

**Fig. 1:**
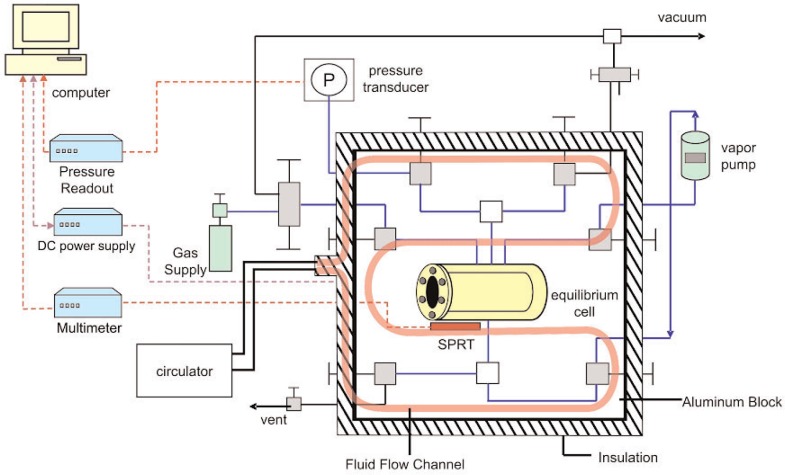
Schematic of the experimental apparatus.

Figure 1 shows a schematic diagram of the vapor-liquid equilibrium apparatus. The heart of the apparatus is the cylindrical equilibrium cell. The cell of 316 stainless steel, has an internal diameter of 2.22 cm and an internal length of 7.62 cm; its internal volume is 30 mL. The cell has a sapphire window at each end to allow visual observation of the fluid being studied. The windows are held in place by bolted flanges and sealed with gaskets on both sides of the windows.

The vapor circulation pump is located outside the aluminum block and is designed to bubble the vapor through the liquid phase. The pump piston is a magnet that is controlled by pulsing power to a solenoid that is wound around the outside of the pump shaft. The pump was included in the apparatus to speed mixing of vapor and liquid phases for measurements of solubility, and thus was not used in the performance test measurements presented here.

Much of the apparatus is contained in a temperature controlled aluminum block. This block consists of two halves, with an overall dimension of 25.4 cm × 30.5 cm × 10.1 cm. The inside faces of each half of the block were machined to accommodate the equilibrium cell, the valves, and the associated connecting tubing. The tolerance of fit for the components in the block is small in order to have all pieces in close thermal contact. The standard platinum resistance thermometer (SPRT) is located in a thermowell alongside the equilibrium cell. Located outside the block are the vapor circulation pump, the heating/cooling circulator, the pressure transducer, and the vacuum pump.

### 2.1 Temperature System

The temperature of the system within the aluminum block was controlled by fluid circulation in conjunction with computer-controlled electric heating (not shown). Flow channels were bored through the sides of the block to allow for the flow of fluid from the temperature-controlled circulator. This fluid flow provided the rough temperature control for the system.

The fine temperature control was achieved with six thin-film heaters (three each, on the large outside faces of both halves of the block). The outside of the block is covered with insulation approximately 7.5 cm thick. In addition, an outside shell of plastic board insulation encompasses the block.

The computer automated temperature control scheme incorporates a proportional-integral-derivative (PID) routine that was developed by Hust et al. [1] and has been shown to be very effective at tight temperature control. The heaters, used in conjunction with the circulator, were capable of maintaining a set block temperature (as measured by the main PRT) within ± 0.005 K indefinitely.

Temperatures are measured with a capsule-type standard PRT (Rosemount model 162D^2^) read by a multimeter (Keithley 2002). The calibration of the standard PRT used in this apparatus was checked against the triple points of both gallium (302.9146 K) and water (273.16 K). The calibration was found to be within ± 0.02 K, but high in both cases. To correct this offset, the *R*_0_ (the thermometer resistance at 273.16 K) value in the calibration was adjusted based on the values recorded during the check of water’s triple point. We estimate the uncertainty in our temperature measurements including the uncertainties in the PRT, voltmeter, and calibration as well as gradients in the aluminum block to be 0.03 K (*K*=2).

### 2.2 Pressure System

Pressure was measured with a commercially available vibrating quartz crystal pressure transducer (Paroscientific model 1001K-01). The manufacturer’s uncertainty specification for this transducer is 0.01 % of full scale. We used a pressure transducer with a range of 0 to 6.89 MPa. Thus the manufacturer’s uncertainty is approximately 7 × 10^−4^ MPa. However, under controlled temperature conditions, and with routine zero adjustment and calibrations, the uncertainty of these types of transducers has been observed to be 0.005 % or less of full scale.

In order to increase the accuracy of our pressure measurement, the pressure transducer was housed in an insulated box that contains a heated aluminum block. The aluminum block is heated with a thin-film heater and the temperature of the box is monitored with a type K thermocouple. The temperature control of the block was maintained with a commercially available controller. The block temperature was maintained at (35.0 ± 0.2) °C (1*σ*).

The pressure transducer used in this work was factory calibrated to primary pressure standards that are traceable to the National Institute of Standards and Technology. The calibration was confirmed in our laboratory using a deadweight pressure gauge (DH Instruments model PG-7601) that is also traceable to a National Institute of Standards and Technology pressure standard. Our in-house calibration showed no significant changes to the factory calibration, except a zero offset of approximately 0.001 MPa. The transducer reading was recorded at vacuum before each set of measurements and reported pressures were corrected to reflect any offset. In addition, a head pressure correction was applied to our results to account for the material in the vertical length of tubing between the cell and pressure transducer. We estimate the uncertainty of our pressure measurements, including the uncertainties in the transducer, calibration and pressure head correction to be 9.8 × 10^−4^ MPa (*K*=2).

### 2.3 Data Acquisition and Measurement Sequence

All instruments were controlled and read by a personal computer over either an RS-232 or an IEEE-488 interface by use of code written in LabVIEW. The measurement sequence included loading the apparatus (under vacuum at 260 K) with the desired fluid. In the case of a pure fluid, the vapor space was evacuated, then partially pressurized to flush out any impurities of lighter weight. This evacuation/pressurization cycle was repeated until the pressure change from one sequence to the next became negligible. The apparatus was considered to be at equilibrium once the temperature had been maintained within ± 0.005 K (outer limit of change) for at least 30 min, and the scatter in the pressure measurements was less than ± 0.01 % (1*σ*). Every 30 s, measurements of the temperature and pressure of the system were recorded. Temperature measurements were made with a multimeter that takes the average of 10 resistance readings from the standard PRT.

### 2.4 Materials

The propane used in these studies was purchased from Scott Specialty Gases and had a stated purity of 99.999 %. This propane was analyzed in our lab with a gas chromatography–mass spectrometry–infrared spectrophotometry method and found to have no major impurities. The butane was purchased from Matheson Gas Products with a stated purity of 99.9 % and was not further analyzed. These two pure gases were used in the preparation of the standard mixture.

### 2.5 Mixture Measurements

In order to study a multi-component system, a gas phase standard mixture of approximately 0.5853/0.4147 mass fraction propane + butane was prepared gravimetrically. The evacuated apparatus was loaded with a small amount of the mixture in the vapor phase and then re-evacuated. This was repeated three times in order to flush the apparatus of any residual contents. The mixture was then condensed into the apparatus, leaving only a very small vapor space. In this way, bubble point measurements on a sample of fixed composition were obtained.

## 3. Results

The results of our vapor pressure measurements of both pure propane and pure butane, and the bubble point measurements of the propane + butane mixture, are listed in Tables 1–3, respectively. Our data are compared to values from the equations of state of Lemmon et al. [2], Buecker and Wagner [3], and Lemmon and Jacobsen [4] for propane, butane, and the mixture, respectively. In addition to the uncertainties in the temperature and the pressure measurement, the combined uncertainty of each vapor pressure measurement was calculated. This value was obtained by correlating the uncertainty in temperature (± 0.03 K) to a pressure uncertainty for each measurement. This was done using the equations of state listed previously. The uncertainty related to the temperature was then combined with the pressure uncertainty (9.8 × 10^−4^ MPa) to give the combined uncertainties (*K*=2) listed in the tables. The effect of sample composition and purity was examined as a contributor to the combined uncertainty of the vapor pressure but was calculated to be negligible.

All three data sets show a similarity, in that the greatest deviations from the respective reference equations are seen at the lowest temperatures. The larger deviations at the lower temperatures are due in part to the higher percentage uncertainty of the pressure measurement at the low end of the range of the pressure transducer, as discussed in the Pressure System section of this paper.

**Fig. 2:**
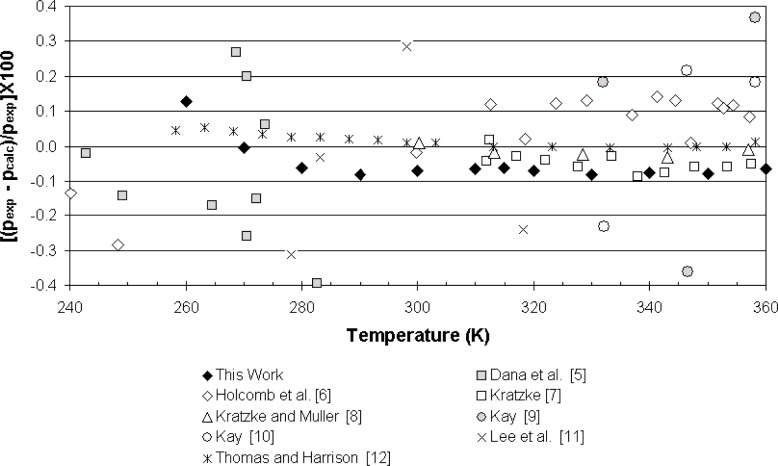
Deviations of vapor pressures of pure propane from the equation of Lemmon et al. [2].

All of the measured data for propane, with the exception of the lowest temperature point, show deviations of less than 0.1 % from values predicted by Lemmon et al. [2]. Figure 2 illustrates the deviations of various data found in the literature from the calculated values of Lemmon et al. [2]. Our data are well within the scatter of these other data sets and agree closely with the data of Kratzke [7] at temperatures of approximately 310 K and above.

**Fig. 3:**
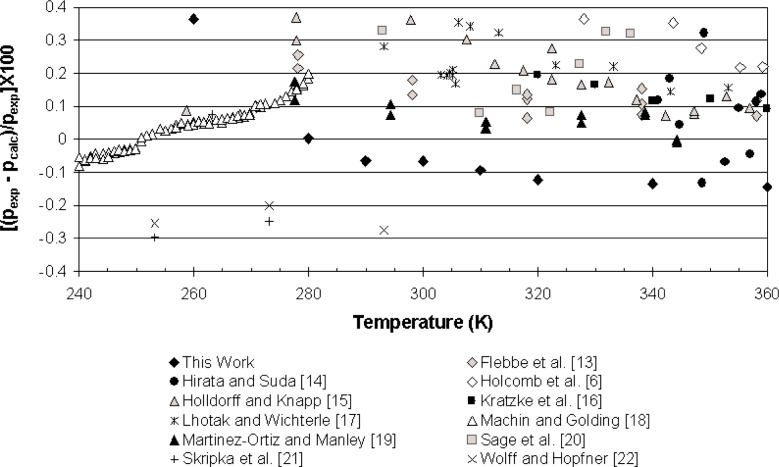
Deviations of vapor pressures of pure butane from the equation of Buecker and Wagner [3].

Figure 3 shows deviations of our data, and literature data sets from the equation of Buecker and Wagner [3] for the vapor pressure of butane. Except for the lowest temperature point, all of our data are within 0.15 % of the correlation. The deviations of our data are primarily negative, while most other data sets show primarily positive deviations. However, the absolute values of the deviations of our data are well within the scatter of the literature data.

**Fig. 4:**
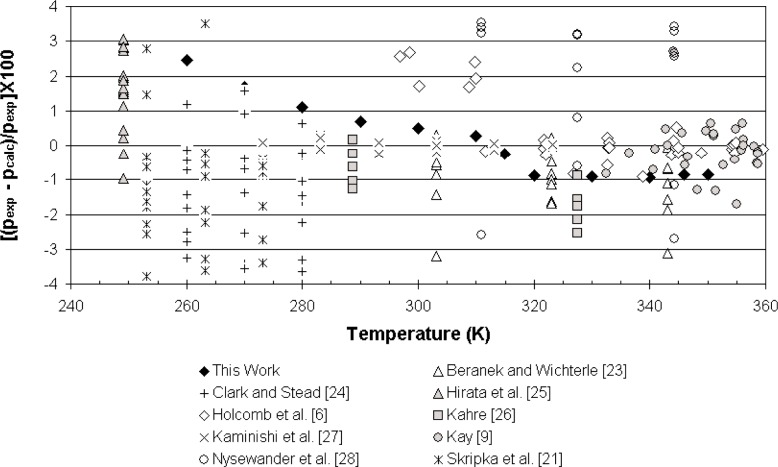
Deviations of bubble point pressures of propane + butane mixtures from the equation of Lemmon and Jacobsen [4].

Figure 4 shows deviations of our data and other propane + butane mixture data from the equation of Lemmon and Jacobsen [4]. In general, uncertainties in mixture data are significantly greater than those for pure fluids due to the added uncertainty in the composition of the mixture. Our data are well within the scatter of data from the literature, particularly at temperatures of 280 K and above.

## 4. Conclusions

Data have been taken over the temperature range of 260 K to 360 K for vapor pressures of pure propane, pure butane, and for bubble points of a mixture of approximately 65 mol % propane and 35 mol % butane. These data demonstrate the capability of our new apparatus for VLE measurements. Our data agree well within the scatter of other experimental data, and the majority of the pure fluid vapor pressures agree within their combined uncertainty to the predictions of the respective equations of state. Future work with this apparatus will include measuring the solubility of gases in ionic liquids.

## Figures and Tables

**Table 1:** Measured vapor pressures of propane and their deviations from the predictions of the equation of state of Lemmon and McLinden [2]

Temperature	Vapor pressure	Combined uncertainty	Percent deviation
(K)	(MPa)	(MPa)	[(exp-calc)/exp]×100
259.981	0.3109	0.0010	0.1271
269.980	0.4301	0.0011	−0.0047
279.979	0.5810	0.0011	−0.0614
289.978	0.7681	0.0012	−0.0800
299.978	0.9965	0.0012	−0.0687
309.977	1.2710	0.0013	−0.0640
314.976	1.4273	0.0014	−0.0618
319.976	1.5970	0.0015	−0.0712
329.975	1.9805	0.0016	0.0807
339.974	2.4284	0.0018	−0.0761
349.973	2.9480	0.0020	−0.0771
359.972	3.5504	0.0022	−0.0651

**Table 2:** Measured vapor pressures of butane and their deviations from the predictions the equation of state of Buecker and Wagner [3]

Temperature	Vapor pressure	Combined uncertainty	Percent deviation
(K)	(MPa)	(MPa)	[(exp-calc)/exp]×100
259.981	0.0612	0.0010	0.3642
279.979	0.1327	0.0010	0.0023
289.978	0.1871	0.0010	−0.0654
299.978	0.2572	0.0010	−0.0670
309.977	0.3457	0.0010	−0.0946
319.980	0.4554	0.0010	−0.1229
339.970	0.7505	0.0011	−0.1351
359.970	1.1680	0.0012	−0.1457

**Table 3:** Measured vapor pressures of propane + butane mixture and their deviations from the predictions of the mixture model of Lemmon and Jacobsen [4]

Temperature	Vapor pressure	Combined uncertainty	Percent deviation
(K)	(MPa)	(MPa)	[(exp-calc)/exp]×100
259.981	0.2262	0.0010	2.4601
269.98	0.3124	0.0010	1.6944
279.979	0.4217	0.0010	1.1119
289.979	0.5574	0.0011	0.6968
299.977	0.7238	0.0011	0.4938
309.977	0.9234	0.0012	0.2804
314.976	1.0324	0.0012	−0.2548
319.976	1.1489	0.0012	−0.8829
329.975	1.4254	0.0013	−0.9231
339.974	1.7472	0.0014	−0.9276
345.973	1.9651	0.0015	−0.8446
349.973	2.1191	0.0015	−0.8671

## Footnotes

2 Certain commercial equipment, instruments, or materials are identified in this paper to foster understanding. Such identification implies neither recommendation or endorsement by the National Institute of Standards and Technology, nor does it imply that the materials or equipment identified are necessarily the best available for the purpose.
